# All biology is computational biology

**DOI:** 10.1371/journal.pbio.2002050

**Published:** 2017-03-09

**Authors:** Florian Markowetz

**Affiliations:** University of Cambridge, Cancer Research UK Cambridge Institute, Cambridge, United Kingdom

## Abstract

Here, I argue that computational thinking and techniques are so central to the quest of understanding life that today all biology is computational biology. Computational biology brings order into our understanding of life, it makes biological concepts rigorous and testable, and it provides a reference map that holds together individual insights. The next modern synthesis in biology will be driven by mathematical, statistical, and computational methods being absorbed into mainstream biological training, turning biology into a quantitative science.

“How do people like you ever get last-author papers?” A leading cell biologist asked me this question in 2008 during the interview for my current job, implying I could never take a senior role in research projects. I had been trained in mathematics and machine learning but was now interviewing for a computational biology job in a cancer research institute. My interviewer wasn’t really sure what my contribution to biology could ever be. Aren’t computational folks just service providers? Handy to have, but without any real scientific vision? She clearly worried about my ability to do independent biological research.

And she was not the last to worry. In 2012, with several last-author papers to my name, I was shortlisted for an European Molecular Biology Organization Young Investigator fellowship but did not get it. The feedback provided by the interview panel called my group a “mathematical service unit,” claimed “a lack of in-depth understanding of biology,” and decried “an overly strong reliance on collaborators.”

Last year, we finally saw how low the opinion of computational work really can be in the biomedical community, when the editor-in-chief of the New England Journal of Medicine used the term “research parasites” to describe computational biologists making sense of published data [1].

Over the last 20 years, computational methods have become a well-established part of biology, but the examples above show that “old school” biologists and clinicians—who make decisions on publications, funding, and careers—continue to be uncomfortable with people like me, who were trained in other disciplines, pursue biological questions different from their own, and use approaches not covered in most biological training. If even my colleagues in the life sciences do not see why computational research matters, how will anybody else be able to see its worth?

In the following, I will argue that computational thinking and computational methods are so central to the quest of understanding life that today all biology is computational biology.

## Computational biology brings order into our understanding of life

“[B]iology adapted itself to the computer, not the computer to biology,” writes Hallam Stevens in *Life Out of Sequence* [2], his ethnographic and historical account of computational biology. He explains: “Computers do not just scale up the old biology, they bring with them completely new tools and questions, like statistics, simulation, and data management, that completely re-shaped the way biological research is being done.”

**Fig 1:**
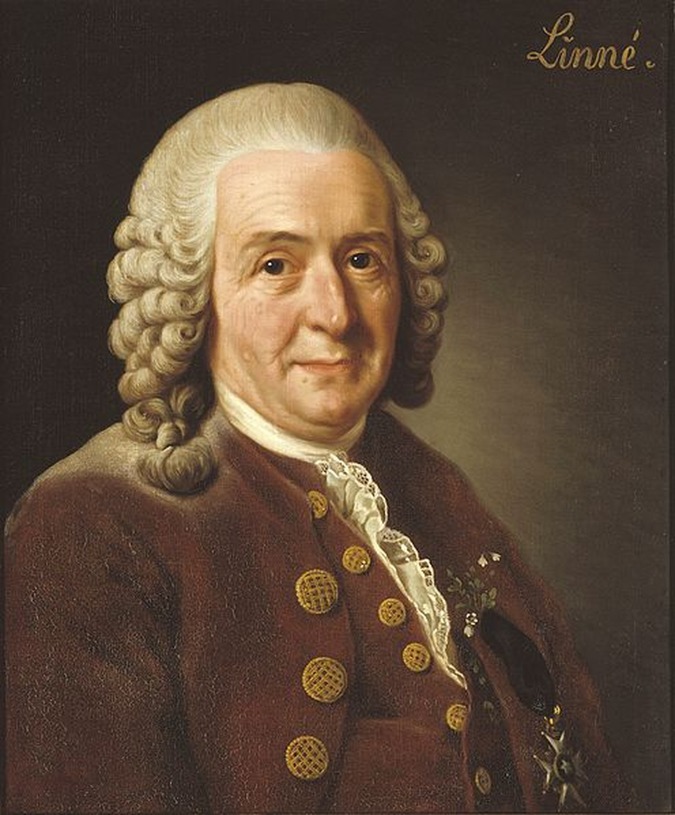
Carl von Linné, the Swedish botanist and father of taxonomy, would be a computational biologist today. Image credit: Nationalmuseum Stockholm.

One key example of how computers reshaped biological research is the use of databases and ontologies. Biological knowledge today is defined, organised, and accessed through computation. If Carl von Linné (also known as Carl Linnaeus), the Swedish botanist and father of taxonomy, lived today, he would be a computational biologist (Fig 1). As a botanist, he might take a leading role in a project like transPLANT (http://www.transplantdb.eu/) to organise what we know about the genotypes and phenotypes of crops and model plants. Or he might work with the Gene Ontology Consortium (http://www.geneontology.org/) to create shared vocabularies that unify biological knowledge across organisms. Just like Linné’s *Systema Naturae*, such databases are key intellectual contributions to our understanding of life. Every other type of biological research builds on these foundations.

## Computational biology lets you see the big picture

Another way computers have reshaped biology is by introducing statistics and data analysis methods. A good example is understanding how mutational processes shape genomes [3]. Mutational processes—be it cigarette smoke, sunlight, or defects in homologous recombination—are not visible in individual mutations but only in their global patterns. How often is a C turned into a T? How does this frequency vary depending on the neighbours of the mutated base? How much of this frequency is explained by other features of the genome, like replication timing? Answering these questions helps us to understand basic properties of the mutational processes active in cells, and it is only possible by statistical techniques that identify patterns and correlations.

These types of analyses need large data collections, and thus the success of computational biology is closely linked to the success of large-scale efforts to gather genotypes and phenotypes of model organisms and humans. One of the first examples highlighting the power of computational approaches was sequencing the human genome, which showed how efficiently computational alignment and scaffolding methods were able to assemble the DNA fragments produced during shotgun sequencing [4], and modern Next Generation Sequencing techniques completely rely on advances in computational biology to analyse huge amounts of short sequence reads [5]. DNA sequencing was once a Nobel Prize–worthy development. Now, computational biology is leading the way in turning it into a widely available and practical approach for both basic biology and medical research, which is currently revolutionising what we know about tissues and single cells.

## Computational biology provides an atlas of life

By combining large data collections with databases and statistics, computational biology is providing a reference map for biology—an atlas of life that holds together individual insights. This map is not at the level of resolution provided by Google Street View, rather, it is a map like the one used by Columbus, Magellan, or Vasco da Gama—intrepid explorers in search of adventure. The map provides a general outline, but many areas are sketchy, and some important parts might even be missing and waiting for discovery. “Here be dragons,” it just says. But even with all these shortcomings, the map is still an indispensable guide: the atlas of life provided by computational biology forms the background for planning, executing, and interpreting all focussed small-scale experiments that probe the uncharted areas and push out the boundaries of biological knowledge.

## Computational biology turns ideas into hypotheses

Finally, computers reshaped biology by making fuzzy concepts rigorous and testable. Here is one example from my own research: for decades, cancer researchers have discussed the idea that genetic heterogeneity between cells in the same tumour helps to make a cancer resistant to therapy [6]. It is a simple idea: the more diverse the cell population is, the more likely it is that a subset of the cells is resistant to therapy and can regrow the tumour after all other cells were killed.

But how exactly can you measure “genetic heterogeneity,” and how big is its influence on resistance development? To answer these questions, we had to turn the idea into a testable hypothesis. We used genomic approaches to measure changes in cancer genomes at different sites in a patient and then defined quantitative measures of heterogeneity, which could be compared statistically to clinical information on treatment resistance. And indeed, we found evidence supporting the initial idea that heterogeneity determines resistance [7].

This is just one of many examples in which a quantitative computational approach was needed to turn a fuzzy idea into a testable hypothesis. Computational biology excels at distilling huge amounts of complex data into something testable in the wet lab, thus, shaping and directing experimental follow-up.

## Rest in peace, computational biology

Pipette biologist. Microscopy biologist. Cell culture biologist. Have you ever heard any of those job titles? No, of course not. All are biologists, because it is the questions you address that matter, not the tools you use, and computational biologists are just biologists using a different tool.

The next modern synthesis in biology will be driven by the absorption of mathematical, statistical, and computational methods into mainstream biological training. It will look more and more like training in physics and combine teaching experimental techniques with mathematical theory and data analysis. And then, even "old school" biologists will view computational biologists as one of their own.
